# Farnesoid X receptor activation protects the kidney from ischemia-reperfusion damage

**DOI:** 10.1038/s41598-017-10168-6

**Published:** 2017-08-29

**Authors:** Zhibo Gai, Lei Chu, Zhenqiang Xu, Xiaoming Song, Dongfeng Sun, Gerd A. Kullak-Ublick

**Affiliations:** 1 Department of Clinical Pharmacology and Toxicology, University Hospital Zurich, University of Zurich, Zurich, Switzerland; 2 Department of Urology, Tengzhou Central People’s Hospital, Zaozhuang, People’s Republic of China; 3 Department of Cardiovascular Surgery, Shandong Provincial Hospital affiliated to Shandong University, Jinan, People’s Republic of China; 4 Department of Thoracic Surgery, Shandong Provincial Qianfoshan Hospital, Shandong University, Jinan, People’s Republic of China

## Abstract

Farnesoid X receptor (FXR) activation has been reported to reduce inflammation and oxidative stress. Because both inflammation and oxidative stress are critical for tissue destruction during kidney ischemia reperfusion (I/R) injury, we investigated the protective role of FXR against kidney damage induced by I/R in mice. Mice undergoing renal I/R developed the typical features of acute kidney injury (AKI): increased creatinine, albuminuria, tubular necrosis and apoptosis. Inflammatory cytokine production and oxidative stress were also markedly increased. In mice pretreated with 6-ethyl-chenodeoxycholic acid (6-ECDCA), a selective FXR agonist, I/R induced changes were prevented and renal function and structure were improved. Moreover, FXR activation also effectively prevented the subsequent progression of AKI to chronic kidney disease (CKD) by ameliorating glomerulosclerosis and interstitial fibrosis and by suppressing fibrogenic gene expression. FXR mRNA levels were inversely correlated with the progression to CKD in mice and with the degree of interstitial fibrosis in human biopsies. In further experiments administering 6-ECDCA to renal proximal tubular cells cultured under hypoxia, the renoprotective effects of FXR activation were associated with inhibition of oxidative and ER stress and with increased antioxidant activity. In conclusion, FXR agonists may have a therapeutic role in conditions associated with ischemic kidney damage.

## Introduction

Acute kidney injury (AKI) is a common complication in patients undergoing major cardiac surgery, those receiving nephrotoxic drugs and those experiencing hemorrhage, dehydration or septic shock^1^. AKI is characterized by an abrupt decrease in the glomerular filtration rate and is a common surgical complication leading to an unacceptably high mortality, chronic kidney disease (CKD) and end stage renal disease (ESRD)^2^. Renal ischemia reperfusion (I/R) injury involves a complex and interrelated sequence of events resulting in renal cell damage and, eventually, cell death by apoptosis and necrosis^3^. Although reperfusion is essential for survival of ischemic tissues, reperfusion itself causes additional cellular injury. This has been attributed to neutrophil infiltration and generation of reactive oxygen species (ROS)^4–6^.

The molecular mechanisms underlying I/R injury are incompletely understood. Diminished oxygen delivery, an accumulation of toxic products in kidney cells and cytokine release play important roles^7, 8^. In addition, ROS generation is critical and is believed to cause tubular epithelial cell damage and death by apoptosis and necrosis^9, 10^. Clinical and experimental studies showed that ROS are crucial for tissue damage and cell apoptosis during I/R, particularly during the process of reperfusion^11–13^. ROS cause lipid peroxidation, disrupting structural integrity and energy production, especially in the proximal tubular segment, which is highly susceptible to acute ischemia and hypoxia^14, 15^. During the process of I/R, the mitochondria and endoplasmic reticulum (ER) are major ROS targets, with outcomes including altered redox homeostasis, mitochondrial damage and protein misfolding in the ER^16^. Several studies closely linked ER stress and mitochondrial dysfunction to the pathogenesis of kidney I/R injury^17^.

Ischemic kidney injury is not always reversible and can lead to ESRD or exacerbate other underlying disease processes, accelerating their progression to end stage kidney failure^18^. In the mouse, a short ischemic episode can lead to persistent interstitial fibrosis^19^ and, in humans, acute ischemic insult occurring immediately after kidney transplantation or cardiovascular bypass may delay recovery of kidney function and lead to chronic nephropathy^20, 21^. Experimental mouse I/R injury has been used extensively to study the pathogenesis of ischemic injury. Although its pathogenesis has become better defined, few therapeutic options are currently available to ameliorate I/R injury^22–25^.

FXR is a member of the nuclear receptor superfamily of ligand-activated transcription factors and functions as an endogenous bile acid sensor^26^. Evidence suggests that the most potent natural activator of human FXR is chenodeoxycholic acid (CDCA). 6-Ethyl-chenodeoxycholic acid (6-ECDCA), with an alkyl substitution that increases its receptor affinity, is a more potent FXR agonist than CDCA^27^. It was recently reported that FXR activation decreased the inflammatory damage and ROS accumulation closely associated with the pathophysiology of renal diseases^28–30^. Therefore, we hypothesized that FXR activation would alleviate renal I/R injury by protecting the mitochondria and ER from ROS and restoring the balance of oxidation and anti-oxidation systems. Our results showed that FXR activation by the FXR agonist 6-ECDCA prevented renal I/R injury, indicating that FXR agonists may have a therapeutic role in conditions associated with ischemic kidney damage.

## Results

### FXR is localized in the nuclei of proximal tubular cells and levels are decreased during I/R damage

**Figure 1:**
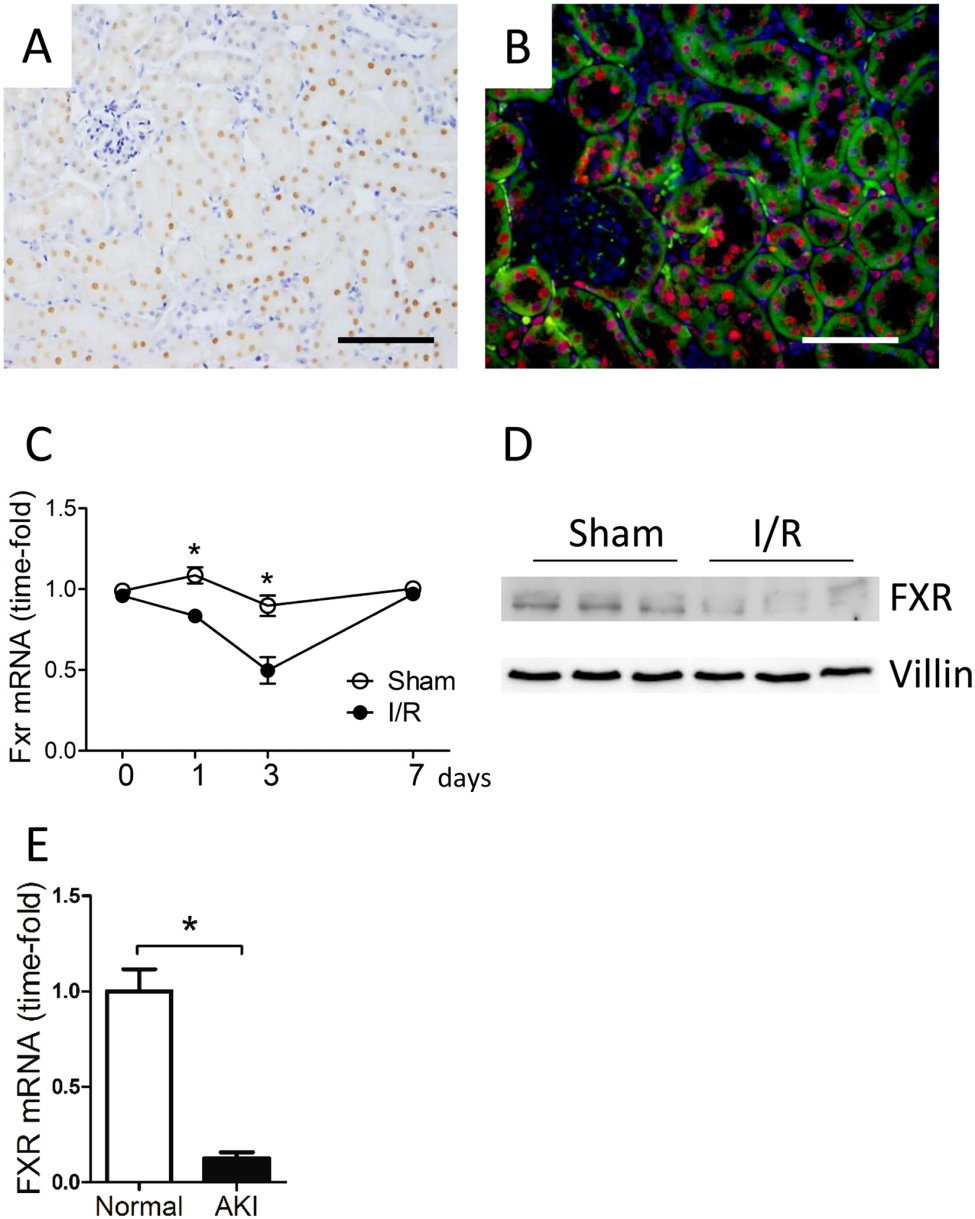
FXR is localized to the proximal tubules of adult mouse kidney cortex and its expression is decreased after ischemia reperfusion. (**A**) Immunohistochemistry for FXR (scale bar 100 μm). FXR was specifically localized to the nuclei of proximal tubular epithelial cells. (**B**) Double immunofluorescence staining of mouse kidneys with antibodies for FXR (red) and villin (green), scale bar 50 μm. Note the extensive co-localization of FXR and villin in the proximal tubules of the cortex. Nuclei were stained with DAPI (blue). (**C**) Levels of FXR mRNA in the kidney from sham and I/R treatment groups at various timepoints. (**D**) Representative western blots showing mouse kidney lysates from the two treatment groups. (**E**) Levels of FXR mRNA from normal and pretransplantation human kidney biopsies. I/R, ischemia reperfusion; AKI, acute kidney injury. *n* = 6 mice/group. Data are means ± SEM, one-way ANOVA with Bonferroni’s test. *p < 0.05.

To examine the precise localization of FXR protein in the kidneys of adult mice and humans, we performed immunostaining for FXR, observing positive staining in the tubular epithelial cells of the cortex region (Fig. 1A). To further characterize FXR localization, an antibody directed against villin, a specific marker for the brush border of proximal tubules, was also used for double immunostaining. The villin positive area was restricted to the cortex regions where there was also FXR staining. FXR was co-localized with villin in proximal tubular epithelial cells (Fig. 1B).

To assess the role of FXR in renal I/R injury, kidney clamping for 30 minutes was followed by reperfusion. Sampling was undertaken at various time points thereafter. We first determined levels of FXR mRNA and protein in renal tissue and found that both were decreased 24 h after reperfusion (Fig. 1C and D). FXR mRNA levels remained decreased for up to 3 d and then returned to normal levels. Similar findings were observed in preimplantation biopsies from individuals with acute ischemic damage (Fig. 1E). The results showed that FXR expression was significantly lower in ischemic kidneys, suggesting that FXR activation was potentially protective during kidney I/R injury.

### FXR activation mitigates I/R injury and improves renal function after reperfusion

We previously reported that FXR activation protects kidney from obesity-induced damage^28^. In exploring the *in vivo* effects of FXR activation, we found that oral and intraperitoneal (i.p.) delivery of 6-ECDCA, an FXR specific agonist, produced very different effects (Fig. S1A). The i.p. injection of 6-ECDCA (5 mg/kg) led to induction of the FXR target gene Shp (Nr0b2) in the kidney, whereas oral administration at the same dosage did not induce Shp expression. To determine the appropriate dosage of 6-ECDCA, we performed a dose response experiment. mRNA expression levels of Shp were induced in the kidney after a single i.p. injection of 6-ECDCA at 5 mg/kg or 10 mg/kg. Our results are consistent with previous studies showing that i.p. injection of an FXR agonist provides more efficient FXR activation in the kidney^31^ and 5 mg/kg of 6-ECDCA are sufficient to activate FXR in the kidney^32^. Unless stated otherwise, 5 mg/kg of 6-ECDCA was used in this study.

**Figure 2:**
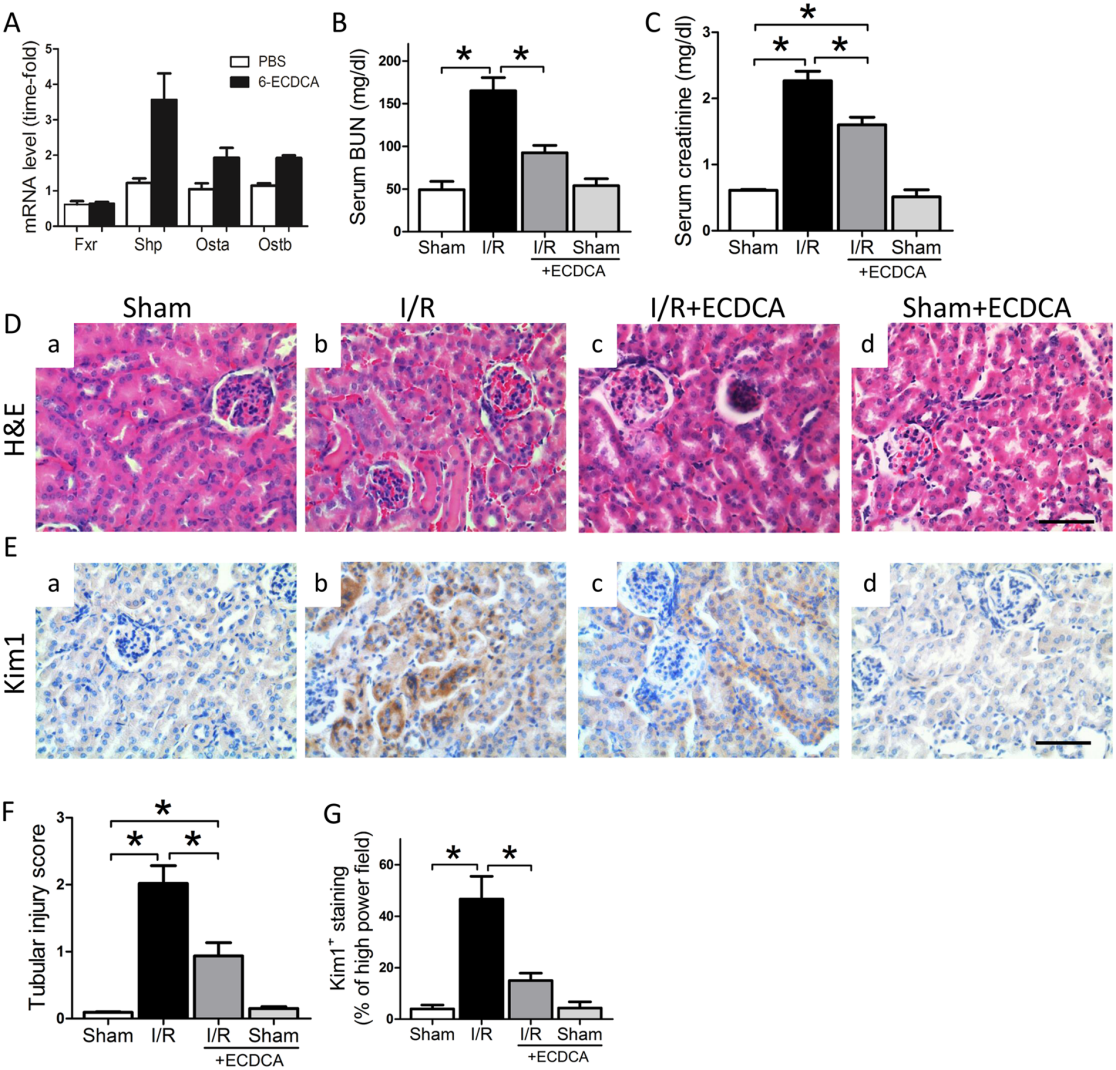
Protection against renal I/R injury by a FXR agonist. Mice were injected i.p. with 6-ECDCA before I/R injury. At 24 h after I/R, kidney, serum and urine samples were collected for measurements of (**A**) FXR target gene expression in kidney, (**B**) BUN in serum, (**C**) creatinine in serum. (**D**) Representative images showing HE staining on renal sections from (a) sham, (b) I/R, (c) I/R + ECDCA and (d) sham + ECDCA groups (scale bar 50 μm). (**E**) Representative images showing Kim1 immunostaining in renal sections from (a) sham, (b) I/R, (c) I/R + ECDCA and (d) sham + ECDCA groups (scale bar 50 μm). Kim1 is specifically localized in the proximal tubules. (**F**) Quantitative analysis of tubular injury scores. *n* = 6 mice/group. (**G**) Quantitative analysis of Kim1 positive staining per high power field. *n* = 6 mice/group. Data are means ± SEM, one-way ANOVA with Bonferroni’s test. *p < 0.05.

Next, to investigate the effects of FXR activation during kidney injury, we injected mice with 6-ECDCA 24 h before inducing ischemia. 6-ECDCA treatment activated FXR sufficiently, as shown by induction of its target genes (Fig. 2A). Renal function was assessed by serum BUN and creatinine. Renal I/R produced a marked increase in serum BUN and creatinine, and all of these effects were attenuated in mice pretreated with 6-ECDCA (Fig. 2B and C). However, serum BUN and creatinine levels in the 6-ECDCA treatment alone group (sham-ECDCA) were identical to the sham group. We next examined how the timing of 6-ECDCA injection affected its protection against I/R. 6-ECDCA injection at either 2 or 8 h before I/R significantly attenuated kidney injury by decreasing serum creatinine (Fig. S2A). In a separate experiment, to examine whether 6-ECDCA had renoprotective effects after the onset of reperfusion injury, we injected the compound 30 min after I/R and observed a small, but significant, decrease in serum creatinine (Fig. S2A). Therefore, 6-ECDCA was most effective when injected before I/R but had a partial benefit when administered after the onset of reperfusion injury. Similar renoprotective effects were observed with GW4064, another FXR agonist. GW4064 treatment significantly attenuated I/R-induced renal dysfunction (Fig. S2B and C). GW4064 also significantly improved the histological scores observed in mice after I/R (Fig. S2B).

Histological analysis showed typical features of severe tubular damage in the I/R group, including loss of the brush border and extensive tubular necrosis (Fig. 2D b). We introduced a scoring system to compare the severity of I/R injury between groups. The tubular injury scores in kidneys subjected to I/R were markedly greater than in those of sham mice (Fig. 2F). Pretreatment with 6-ECDCA preserved the normal morphology of the kidney and resulted in only a slight loss of the brush border. In addition, the 6-ECDCA pretreated mice also had less severe tubular necrosis (Fig. 2D c). Quantification of tubular injury by immunostaining for Kim-1 correlated with renal function and histological findings. As compared with the sham mice, the kidneys of I/R mice showed extensive Kim-1 staining in the proximal tubules (Fig. 2E b and G). However, with 6-ECDCA pretreatment there was negligible Kim-1 staining in the tubules (Fig. 2E c). Both histological analysis and Kim-1 staining results in the 6-ECDCA treatment alone group (sham-ECDCA) were identical to the sham group (Fig. 2D d and E d, respectively), consistent with serum BUN and creatinine levels (Fig. 2B and C). Therefore, we focused on the comparison between the I/R group and the I/R + ECDCA group in the subsequent studies.

### FXR activation decreases inflammation and apoptosis in I/R injured kidney tissue

**Figure 3:**
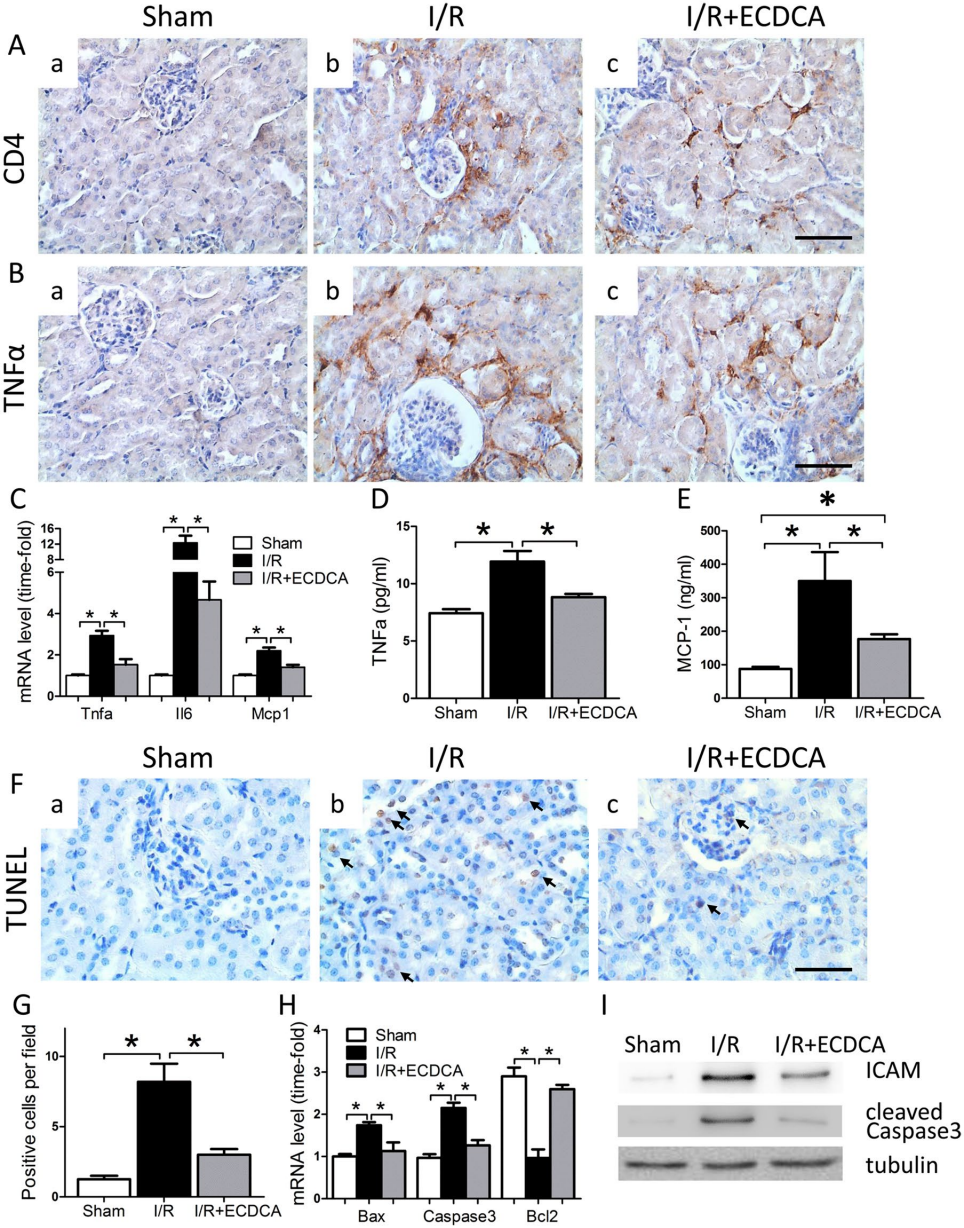
Decreased inflammation, inflammatory mediator production and apoptosis with 6-ECDCA pretreatment. (**A** and **B**) Representative images of immunostaining for (**A**) CD4 and (**B**) TNFα in renal sections from (a) sham, (b) I/R and (c) I/R + ECDCA groups (scale bar 50 μm). Note the tubulointerstitial localization of CD4^+^ and TNFa^+^ inflammatory cells. (**C**) Levels of mRNA for Tnfα, Il6 and Mcp1 in kidney tissue. *n* = 6 mice/group. (**D** and **E**) Blood levels of (**D**) TNFα and (**E**) MCP1. *n* = 6 mice/group. (**F**) Representative images of TUNEL staining on renal sections from (a) sham, (b) I/R and (c) I/R + ECDCA groups (scale bar 50 μm). Arrows indicate apoptotic bodies. (**G**) Quantitative analysis of positive TUNEL stained cells per high power field. *n* = 6 mice/group. (**H**) Levels of Bax, Caspase3 and Bcl2 mRNA in kidney tissue. *n* = 6 mice/group. Data are means ± SEM, one-way ANOVA with Bonferroni’s test. *p < 0.05. (**I**) Western blot analysis of ICAM and cleaved-caspase 3 protein levels in kidney samples of sham, I/R and I/R + ECDCA mice.

The inflammatory response following I/R injury is known to be related to renal dysfunction^15^. To investigate whether the improved renal function in 6-ECDCA pretreated mice was dependent on a suppressed inflammatory reaction, we stained kidney tissues for CD4 and tumor necrosis factor alpha (TNFα). After 24 h reperfusion, the number of CD4^+^ cells was increased in kidneys of mice that had been subjected to only I/R (Fig. 3A b). Levels of the inflammatory cytokine TNFα were also increased in these kidneys (Fig. 3B b). However, staining for both CD4 and TNFα was significantly decreased in 6-ECDCA pretreated mice (Fig. 3A c and 3B c, respectively). Real-time PCR analysis confirmed that the 6-ECDCA pretreated I/R group had less inflammation than the I/R group (Fig. 3C). Changes in serum TNFα and MCP-1 levels were consistent with the changes of TNFα and MCP-1 mRNA levels, which were significantly increased in the I/R kidney and ameliorated by 6-ECDCA treatment (Fig. 3D and E). ICAM, a cell surface glycoprotein induced by interleukin-1 and TNFα in leukocytes, was increased in the I/R kidneys. However, this increase was attenuated by FXR activation (Fig. 3I).

Apoptosis is a major mediator of tubular cell death after renal I/R injury. To investigate whether the protective effect of FXR activation in renal injury involved decreased apoptosis, the extent of kidney apoptosis was evaluated by TUNEL staining (Fig. 3F). The number of TUNEL-positive apoptotic cells was markedly greater in I/R mice than in sham mice (Fig. 3F b, indicated by arrows, and 3 G). After I/R, the kidneys also had increased levels of proapoptotic caspase-3 and Bax, decreased mRNA for the anti-apoptotic factor Bcl2 (Fig. 3H) and increased cleaved-caspase-3 protein level (Fig. 3I). Pretreatment with 6-ECDCA attenuated these changes (Fig. 3F c, H and I).

### FXR activation attenuates renal oxidative and ER stress after I/R

**Figure 4:**
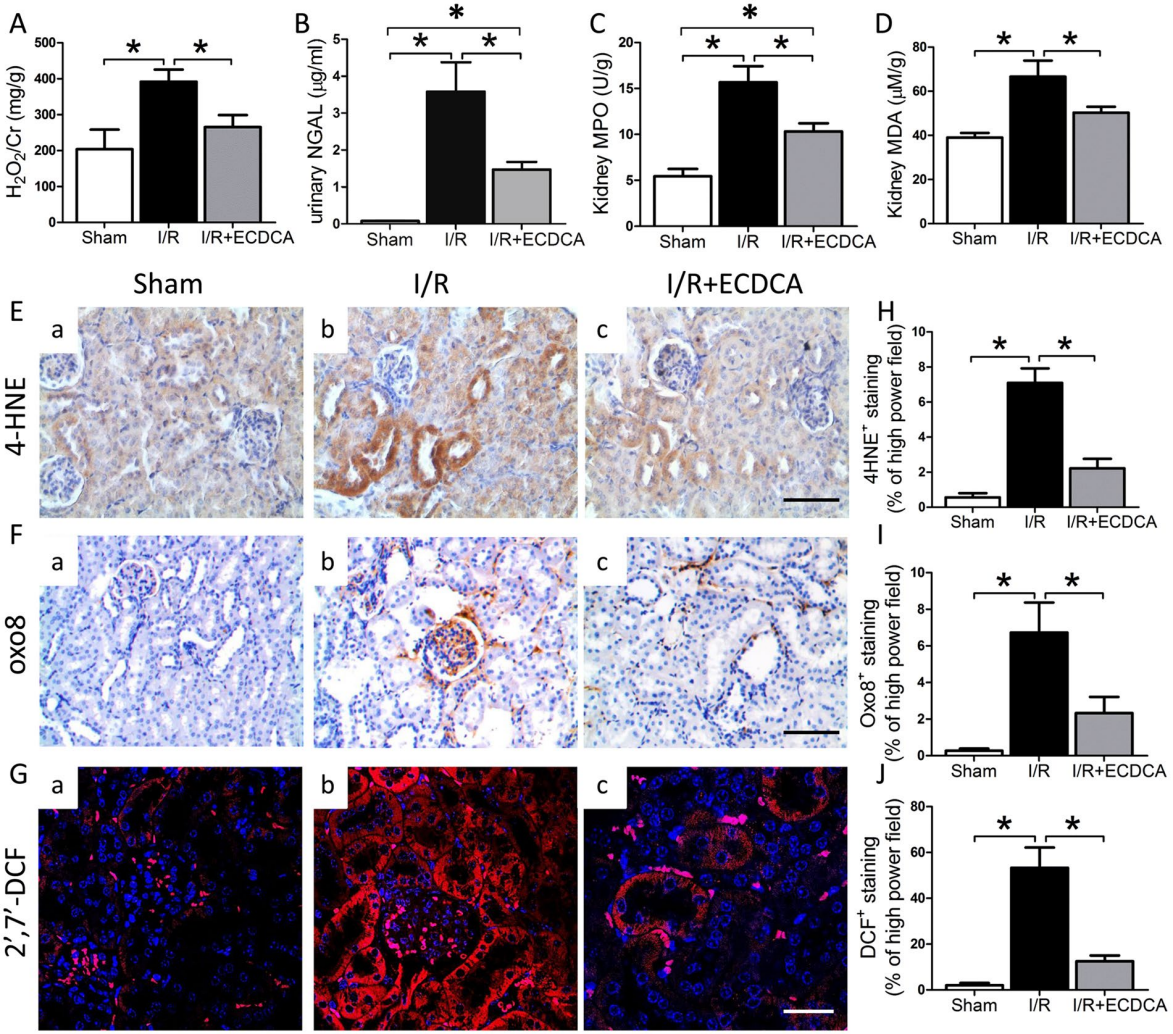
Attenuation of I/R-induced oxidative stress by 6-ECDCA treatment. At 24 h after treatment, (**A**) urinary H_2_O_2_, (**B**) urinary NGAL, (**C**) kidney MPO and (**D**) kidney MDA were analyzed. *n* = 6 mice/group. Data are means ± SEM, one-way ANOVA with Bonferroni’s test. *p < 0.05. (**E** and **F**) Representative images of immunostaining for (**E**) 4-HNE and (F) oxo-8 staining on renal sections from (a) sham, (b) I/R and (c) I/R + ECDCA groups (scale bar 50 μm). (**G**) 2′,7′-DCF staining on renal sections from (a) sham, (b) I/R and (c) I/R + ECDCA groups (scale bar 25 μm). (**H**–**J**) Quantitative analysis of (H) 4-HNE, (I) oxo-8 and **(J**) 2′,7′-DCF staining per high power field. *n* = 6 mice/group. Data are means ± SEM, one-way ANOVA with Bonferroni’s test. *p < 0.05.

Oxidative stress and lipid peroxidation after I/R induce mitochondrial and ER stress. We first investigated whether FXR activation decreased oxidative stress in kidneys subjected to I/R by measuring several oxidative stress indicators, urinary H_2_O_2_ and neutrophil gelatinase-associated lipocalin (NGAL) and kidney myeloperoxidase (MPO) and MDA. Compared with the sham group, I/R significantly increased all these indicators (Fig. 4A–D). The levels of these indicators were lower, however, with 6-ECDCA pretreatment, suggesting that FXR activation had antioxidant effects. 4-HNE formation and deoxyguanosine oxidation are common chemical markers of increased ROS. We detected 4-HNE and 8-oxo-dG by immunostaining (Fig. 4E and F) and found that both were increased in I/R compared with sham kidneys (Fig. 4E b and F b). 6-ECDCA pretreatment suppressed ROS production in kidneys subjected to I/R, as indicated by decreased staining for 4-HNE and 8-oxo-dG (Fig. 4E c and F c). Quantitative analysis of 4-HNE and 8-oxo-dG stained cells confirmed decreased ROS production in I/R kidneys, and staining for both markers was attenuated by 6-ECDCA pretreatment (Fig. 4H and I). To confirm these results, we examined ROS production using H_2_DCFDA, a specific fluorescence indicator for ROS. Upon cleavage of the acetate groups by intracellular esterases, followed by ROS-induced oxidation, the nonfluorescent H_2_DCFDA is converted to the highly fluorescent 2′,7′-dichlorofluorescein (2′,7′-DCF). The fluorescence in renal tissue exposed to I/R was about 50-fold higher than in controls (Fig. 4G b and J). The clearest changes in ROS production occurred in tubule cells (Fig. 4G b). 6-ECDCA pretreatment resulted in a remarkable decrease in DCF fluorescence after I/R, indicating less oxidative stress (Fig. 4G c and J).

**Figure 5:**
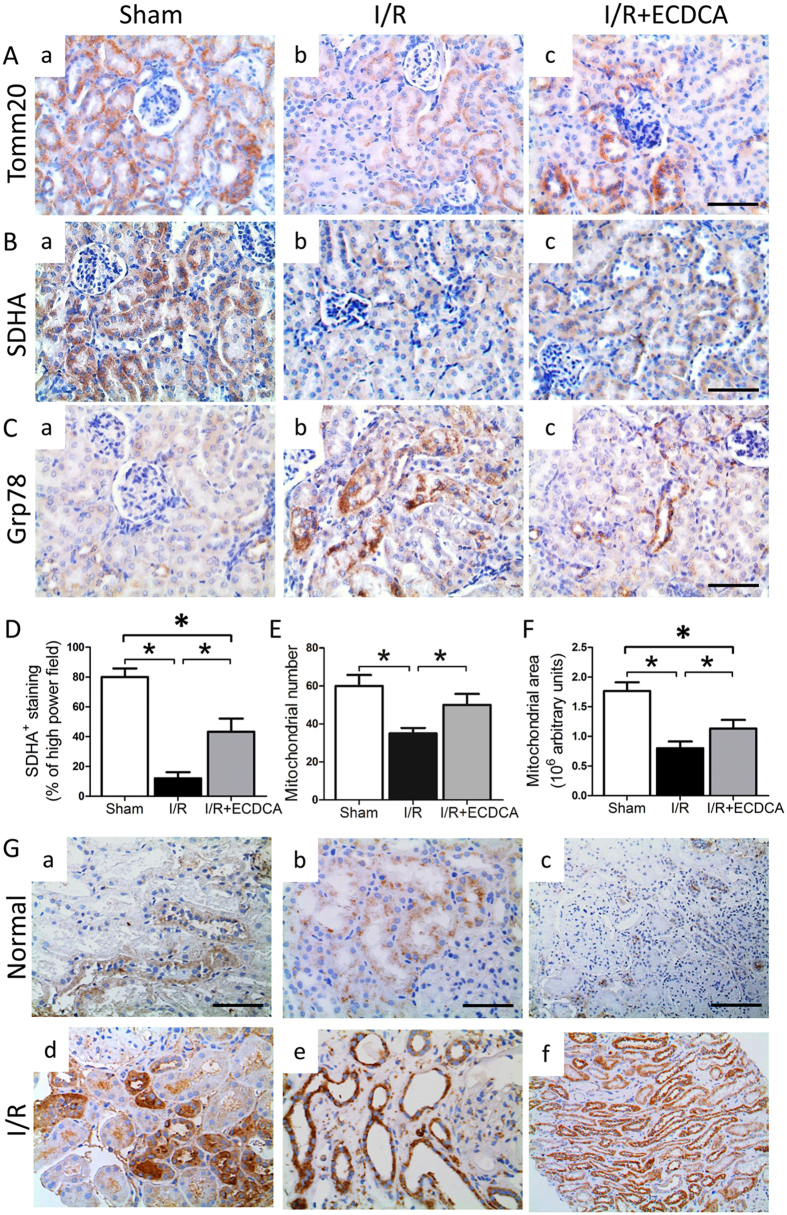
FXR activation protects mitochondrial function and structure and inhibits ER stress during I/R-induced injury. (**A**–**C**) Representative images showing immunostaining for (**A**) Tomm20, (**B**) SDHA and (**C**) Grp78 on renal sections from (a) sham, (b) I/R and (c) I/R + ECDCA groups (scale bar 50 μm). Note all three protein markers are localized in proximal tubules. (**D**–**F**) Quantitative analysis of (**D**) SDHA staining per high power field, quantitative analysis of (**E**) mitochondrial number and (**F**) mitochondrial area. *n* = 6 mice/group. Data are means ± SEM, one-way ANOVA with Bonferroni’s test. *p < 0.05. (**G**) Markers for oxidative and ER stress in normal and preimplantation human renal biopsy specimens. Shown are representative images of immunostaining for (a and d) 4-HNE, and (b and e) GRP78 in kidney specimens obtained from (a and b) normal biopsies and (d and e) preimplantation biopsies (magnification 400x). (c and f) CHOP in kidney specimens obtained from (c) normal biopsies and (f) preimplantation biopsies (scale bar 100 μm).

Immunostaining for Tomm 20 and SDHA, markers for mitochondria and for mitochondrial respiratory chain function, indicated a decreased number of mitochondria and a decreased function of the mitochondrial respiratory chain in I/R kidneys (Fig. 5A b and B b). I/R also led to decreased kidney levels of SDHA and Nrf2 proteins, detected by western blotting (Fig. S3). This was consistent with electron microscopy, which showed decreased mitochondrial numbers, as well as rounded and swollen mitochondria, after I/R (Fig. 5E and F). In contrast, mitochondrial numbers and their architecture after I/R were closer to normal with FXR activation (Fig. 5D–F). Immunostaining for Grp78, a marker for ER stress, indicated ER dysfunction in the kidneys of I/R-injured mice (Fig. 5C b). However, ER stress was attenuated in mice pretreated with 6-ECDCA (Fig. 5C c and G). This indicated that FXR activation was protective against I/R-induced ER stress.

To translate these findings into the clinical setting, we measured markers of oxidative and ER stress in patient biopsies from kidneys that had undergone acute ischemic damage (preimplantation biopsies). Immunostaining for 4HNE, GRP78 and CHOP produced similar findings to those obtained in the murine I/R model (Fig. 5G a-f). Staining for the three markers was predominant in the tubules of kidneys with acute ischemic damage, compared with normal kidneys, indicating that increased oxidative and ER stress were induced by I/R in deceased kidney donors during kidney transplantation procedures.

### FXR activation prevents CKD in mice at 4 months after I/R

**Figure 6:**
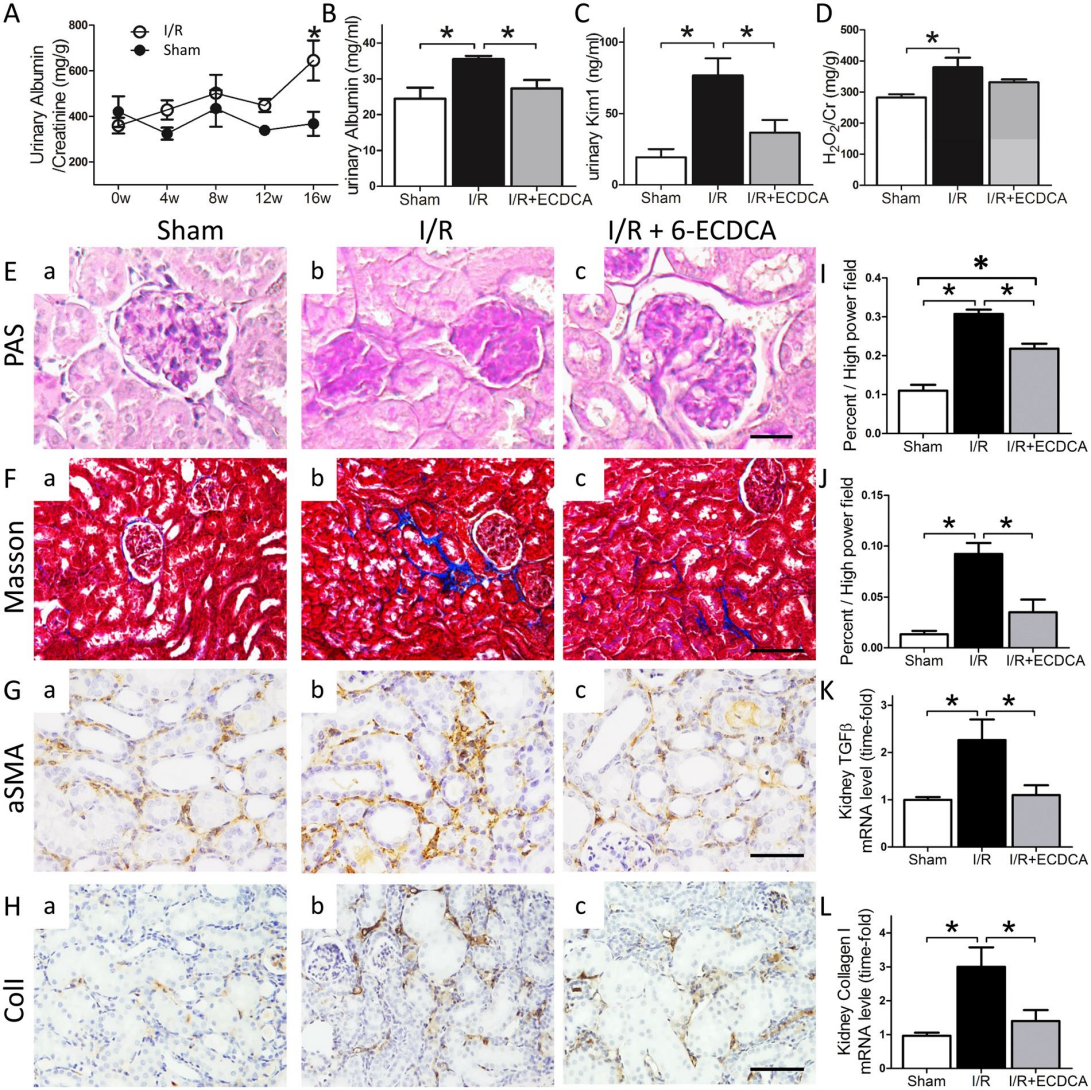
Prophylactic 6-ECDCA administration prevents progression to CKD after an I/R induced AKI episode. (**A**) Urinary albumin/creatinine ratios measured every 4 wk after surgery in sham and I/R groups. (**B**) Urinary albumin, (**C**) Kim1 and (**D**) H_2_O_2_ levels from different treatment groups. *n* = 6 mice/group. Data are means ± SEM, one-way ANOVA with Bonferroni’s test. *p < 0.05. (**E**) Representative images of PAS staining (scale bar 25 μm). (F-H) Representative images of (**F**) Masson’s trichrome staining and immunostaining for (**G**) αSMA and (**H**) collagen I in renal sections from (a) sham, (b) I/R and (c) I/R + ECDCA groups (scale bar 100 μm). αSMA and collagen I are localized in the tubulointerstitial spaces as markers for tubulointerstitial fibrosis. (**I** and **J**) Bars represent quantitative analysis of the **(I**) mesangial area and (**J**) fibrosis area per high power field. (K and L) Bars represent (**K**) TGF-β and (**L**) collagen I mRNA levels in the kidneys. *n* = 6 mice/group. Data are means ± SEM, one-way ANOVA with Bonferroni’s test. *p < 0.05.

In a separate experiment, animals were monitored for 16 wk after I/R, with or without 6-ECDCA pretreatment, for comparison with their respective control groups. As shown in Fig. 6A, during the first 2 mo after AKI recovery, none of the mice subjected to I/R exhibited proteinuria. However, at 4 mo after I/R, there were higher levels of urinary protein in the I/R group compared with sham group (Fig. 6B). At the end of the experimental period, the I/R group had significantly increased urinary biomarkers Kim-1 and H_2_O_2_ (Fig. 6C and D). These renal functional changes were not observed in the I/R group that had been pretreated with 6-ECDCA. Histopathologically, the I/R group had severe structural damage comprising glomerulosclerosis and interstitial fibrosis, effects confirmed by PAS and Masson’s trichrome staining respectively (Fig. 6E b and F b). However, the 6-ECDCA pretreated group appeared to be protected from such pathological changes (Fig. 6E c, I, F c and J). Activation of TGF-β and fibrogenic genes is believed to play a role in the progression to CKD. TGF-β and collagen I mRNA levels were increased in the I/R group (Fig. 6K and L). Immunostaining for αSMA and collagen I was increased in the I/R injured kidney (Fig. 6G and H). This staining pattern was not observed in the 6-ECDCA pretreated group, with only minor fibrosis evident in these mice.

**Figure 7:**
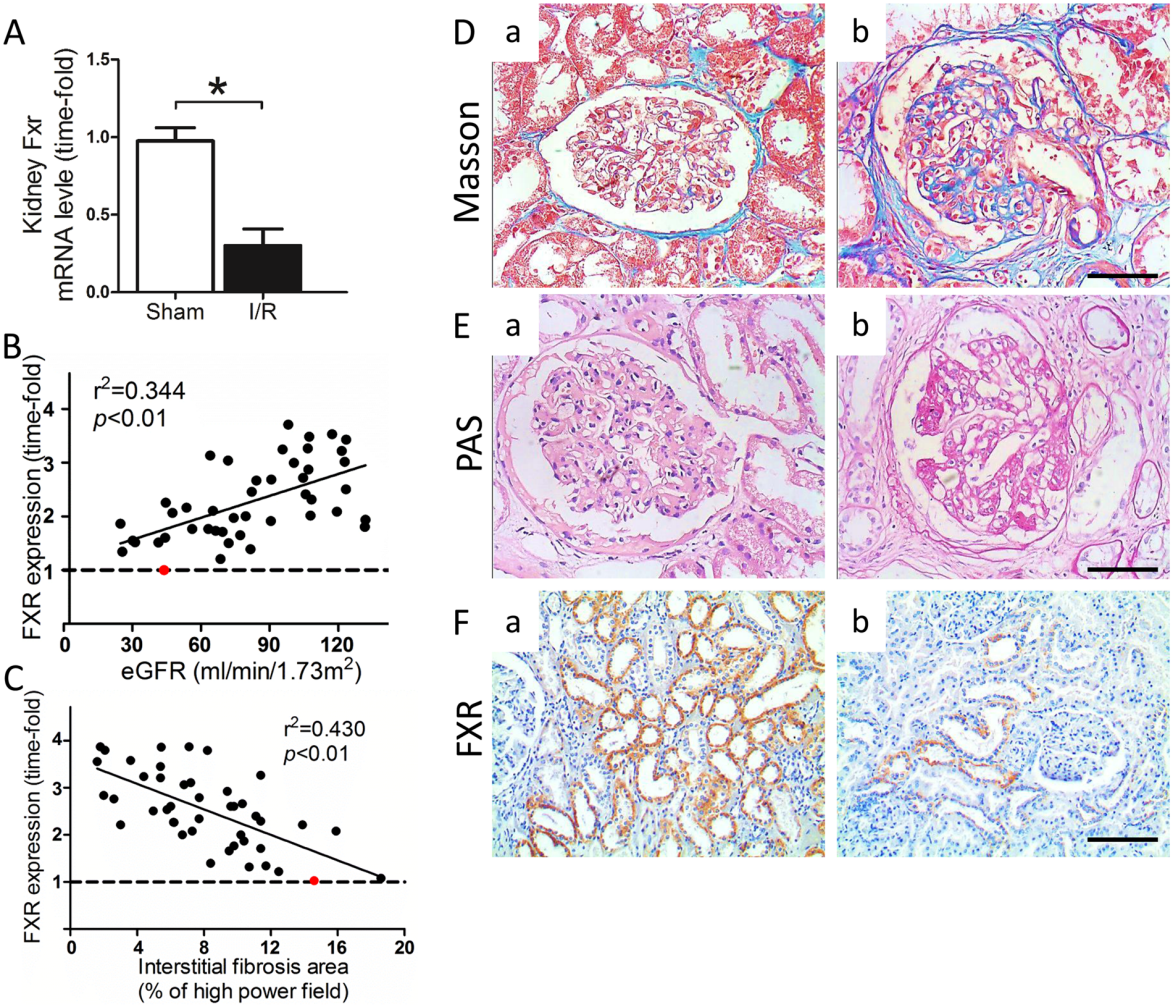
FXR mRNA expression is inversely correlated with CKD progression. (**A**) FXR mRNA expression in kidney samples from sham and I/R mice 16 wk after surgery. (**B**) FXR mRNA expression in human kidney biopsies was directly correlated with eGFR. (**C**) FXR mRNA expression in human kidney biopsies was inversely correlated with interstitial fibrosis, as determined by histology. The lowest level of FXR mRNA expression was set to 1 (shown as ). (**D** and **E**) Representative images of (**D**) Masson’s trichrome staining and (**E**) PAS staining in renal biopsy sections from (a) normal subjects and (b) patients with CKD (scale bar 20 μm). (**F**) immunostaining for FXR in renal biopsy sections from (a) normal subjects and (b) patients with CKD (scale bar 100 μm).

Reduced FXR expression levels have been related to CKD progression. At 4 mo after I/R induced injury, FXR expression at the mRNA level was decreased (Fig. 7A). This decrease was confirmed by findings in kidney biopsies of patients after cardiac surgery. We examined 25 kidney biopsies from patients who had developed CKD after cardiopulmonary bypass, compared with normal controls. Details are summarized in Supplementary Table S1. Histological staining for fibrosis and glomerulosclerosis showed that these patients had CKD (Fig. 7D and E). The intensity of FXR staining was lower than in patients who did not have kidney damage (Fig. 7F, b compared with a). eGFR values were directly correlated with FXR mRNA levels in the kidneys (r^2^ = 0.401, Fig. 7B). Moreover, the incidence of interstitial fibrosis was significantly higher in patients with low FXR mRNA levels (r^2^ = 0.286, Fig. 7C), indicating a negative correlation between FXR expression and CKD progression.

### FXR activation increases cell viability and reduces cellular oxidative stress and apoptosis

Next, we examined whether FXR expression was decreased in proximal tubular cells exposed to hypoxia. As shown in Fig. S4A, the level of FXR protein detected by immunoblotting was significantly lower in cells exposed to hypoxia/re-normoxia (H/R) than in those cultured under normoxia. FXR mRNA measurements confirmed its decreased expression in hypoxic cells (Fig. S4B).

**Figure 8:**
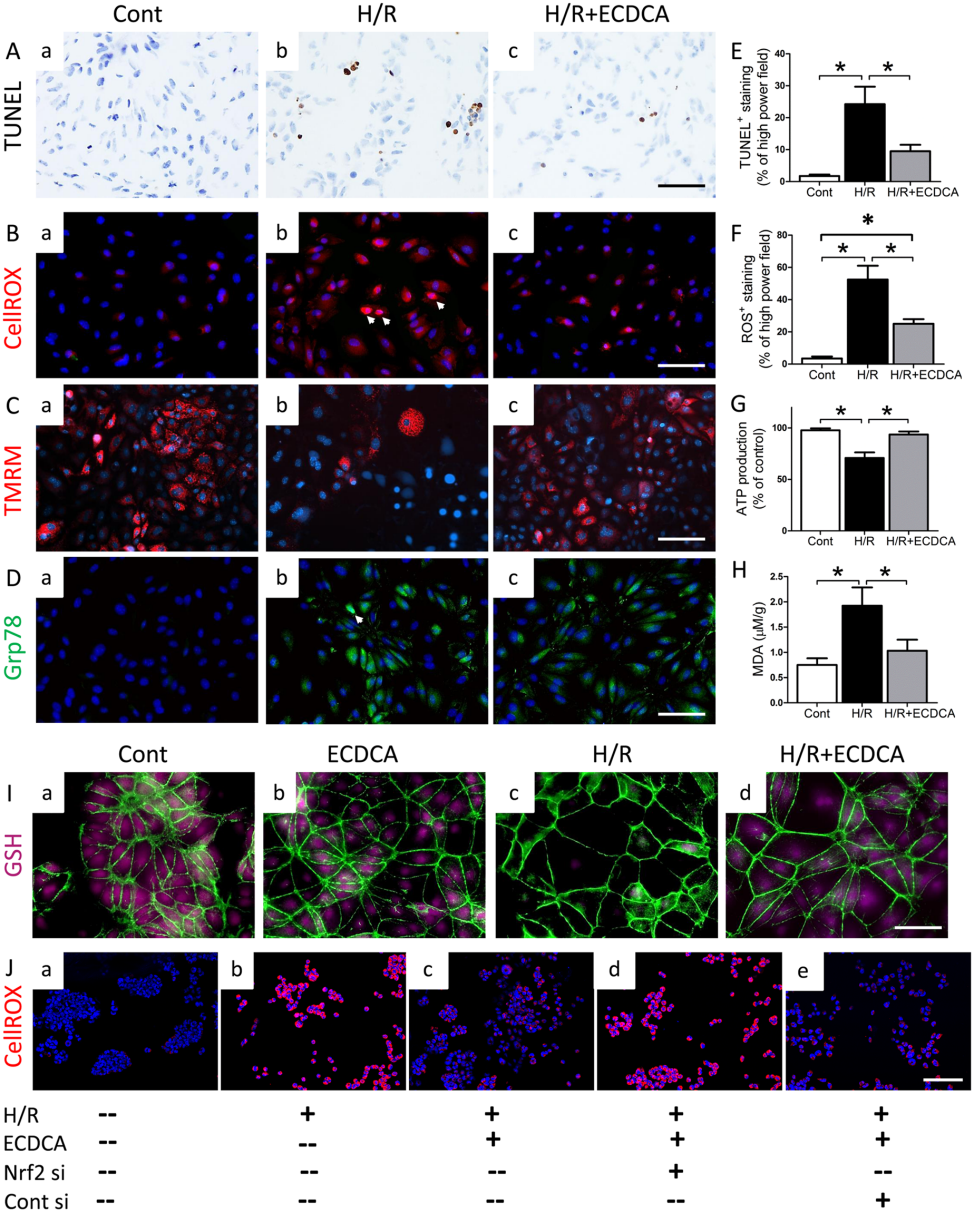
Effects of FXR activation on hypoxia-induced oxidative stress in primary cultured proximal tubular cells (PTCs). H/R, hypoxia/re-normoxia. (**A**–**D**) Representative images of (**A**) TUNEL staining, **(B**) CellROX staining, (**C**) TMRM staining and (**D**) immunostaining for Grp78 on PTCs treated with vehicle under (a) normoxia and at 24 h after exposure to hypoxia (b) without 6-ECDCA treatment or (c) with 6-ECDCA treatment (scale bar 50 μm). Staining colors correspond to the labels on the left side of each panel. Cells were counterstained with hematoxylin (blue) or DAPI (blue). (**E**–**H**) Quantitative analysis of positive (**E**) TUNEL staining and (**F**) ROS staining per high power field and (**G**) ATP production and (**H**) intracellular MDA in PTCs. *n* = 4/group. Data are means ± SEM, one-way ANOVA with Bonferroni’s test. *p < 0.05. (**I**) Representative images of GSH detection in PTCs with different treatments (scale bar 50 μm). H/R, hypoxia/re-normoxia. Staining colors correspond to the labels on the left side of each panel. Cells were counterstained with ActinGreen (green). (**J**) Representative images of CellROX staining of PTCs treated as indicated (scale bar 100 μm).

To better assess the role of FXR activation in preventing I/R induced kidney damage, we cultured primary proximal tubular cells from mouse kidney and exposed them to hypoxia, with or without FXR agonists. 6-ECDCA treatment successfully activated FXR, as indicated by increased mRNA expression of the FXR-activated genes Shp, Ostα and Ostβ (Fig. S4C). Similar results were also obtained in cells treated with GW4064 (Fig. S4C). Next, we examined the effects of FXR activation on cell viability. Hypoxia decreased cell viability by about 20%, compared with control cells (Fig. S4D). Pretreatment with 6-ECDCA at both low (1 µM) and high (4 µM) concentrations increased cell survival during hypoxia, consistent with TUNEL staining and quantitative TUNEL analysis (Fig. 8A c, B, and S4 D).

Exposure of cells to hypoxia also resulted in oxidative stress, mitochondrial damage and ER stress, as denoted by significantly higher signals in assays for intracellular ROS, TMRM, Grp78 and MDA, respectively (Fig. 8B b, C b, D b and H). Notably, FXR activation in proximal tubular cells resulted in markedly decreased oxidative stress, mitochondrial damage and ER stress in response to hypoxia (Fig. 8B c, C c, D c, H and Fig. S4A). Quantitative analysis of ROS-dependent signals under various treatments confirmed decreased ROS levels in 6-ECDCA treated cells (Fig. 8F). Changes in levels of ATP and SDHA protein (Fig. 8G and Fig. S4A), both markers of intact mitochondrial function, were consistent with the results of TMRM staining. ATP and SDHA protein levels were lower in cells exposed to hypoxia compared with normoxia, and both effects were attenuated by 6-ECDCA treatment (Fig. 8G and Fig. S4A). This indicated that FXR activation promoted recovery of mitochondrial function in cells exposed to hypoxia.

### 6-ECDCA attenuates hypoxia-induced oxidative stress through induction of Nrf2-mediated antioxidant effects

We previously showed that FXR activation decreased oxidative stress by inducing antioxidant genes in proximal tubular cells^28^. To confirm this finding, we investigated intracellular glutathione (GSH) levels in cells subjected to various treatments. Hypoxia resulted in a 30% decrease in intracellular GSH (Fig. 8I c and Fig. S5A). Co-treatment of cells undergoing hypoxia with 6-ECDCA attenuated this GSH decrease (Fig. 8I d and Fig. S5A). Levels of mRNA for antioxidant genes were also examined in cells exposed to hypoxia. Hypoxia decreased mRNA expression of Acox1, Gclm, Gpx1 and Nqo1, all of which were less suppressed in cells also co-treated with 6-ECDCA (Fig. S5B). In addition, GSH levels and mRNA expression of Gclm, Sod2 and Nqo1 in the kidney cortex of 6-ECDCA treated I/R mice were also increased (Fig. 8K and Fig. S6A to C). Recent studies demonstrate the interactions between the FXR signaling pathway and Nrf2-mediated antioxidant effects^33, 34^. Therefore, we examined whether the protective effects of FXR activation and the induction of antioxidant genes were mediated through Nrf2. 6-ECDCA reduced hypoxia-induced oxidative stress in the PTCs (Fig. 8J c). When the expression of Nrf2 is knocked down, 6-ECDCA failed to exert its effect on the redox state (Fig. 8J d), indicating a major role of Nrf2 in mediating the inhibitory effect of FXR activation on the oxidative state. As shown by western blot analysis, FXR activation induced Nrf2 protein levels in the nuclei of PTCs treated with 6-ECDCA (Fig. S7). Silencing of FXR in PTCs transfected with FXR siRNA blocked this induction (Fig. S7). Consistent with this, immunostaining for Nrf2 showed Nrf2 translocation in the proximal tubule cells of mouse kidney with 6-ECDCA treatment (Fig. S8 C and F respectively). Moreover, both Nrf2 and FXR siRNA blocked the induction of Gclm, a target gene of Nrf2 (Fig. S9). Therefore, FXR activation protected kidney proximal tubular cells by inducing Nrf2-mediated antioxidant pathways.

## Discussion

In this study, we demonstrated that pretreatment with the FXR agonist 6-ECDCA protected against renal I/R injury. After I/R, 6-ECDCA pretreated mice displayed significantly better renal function, less histological tubular damage and reduced inflammation, compared with those subjected to I/R alone. These protective effects were associated with ameliorated oxidative stress, as manifested by an increased antioxidant capacity, that is, higher GSH and lower ROS levels. Consistent with our findings in mice, our analysis of human renal biopsy samples showed greater oxidative and ER stress in kidneys that had undergone ischemia. Moreover, FXR expression was negatively correlated with the progression of tubular interstitial fibrosis and CKD, observed at longer time periods after ischemic damage. Thus, FXR activation might represent a novel protective strategy against I/R-induced kidney damage over both short and long time periods.

Repression of FXR was reported to contribute to biliary injury in liver grafts^35^. *In vitro* data suggested that hypoxia downregulated FXR via a p38MAPK dependent pathway^36^. FXR expression was inversely correlated with the progression of obesity and diabetic nephropathy^37, 38^. It was also reported that the FXR signaling pathway was repressed during kidney I/R injury^39^. Consistent with these reports, our data showed that FXR expression was substantially decreased during the early period after I/R and that its expression level was inversely correlated with CKD progression. The beneficial effects of FXR activation in modulating kidney injury were demonstrated in our previous studies in obese mice, using obeticholic acid^28^. FXR activation proved to be beneficial against obesity and diabetes-induced kidney damage because of its antioxidant effects. The role of FXR in modulating lipid metabolism and renal disease was also demonstrated in STZ diabetic mice, mice with diet-induced obesity and db/db mice^40–42^. Our present findings not only showed involvement of FXR in I/R induced injury but also revealed a more profound role for FXR in renal pathophysiology related to oxidative stress.

In our study, CKD was induced using a single episode of AKI in mice and we obtained evidence that an early intervention, administration of 6-ECDCA, was important for preventing or slowing CKD progression. Our data showed that FXR activation effectively prevented the transition to CKD. The I/R group developed CKD, characterized by renal dysfunction, glomerulosclerosis and tubulointerstitial fibrosis (Fig. 7). It was previously believed that patients recovering from an AKI episode experienced no further deterioration of kidney function. However, AKI is now known to be an independent risk factor for development of CKD and transition from CKD to end-stage renal disease^43–45^. It was estimated that 20% of patients experiencing an episode of AKI would develop CKD within 3 years^46^. Recent studies showed that progression to CKD from ischemia-induced AKI depended on the extent of tubular epithelial death mediated by the TNFα pathway^47^ and on the sustained activation of Wnt/beta-catenin signaling^48^. These abnormalities certainly accelerated deterioration of the functional nephrons. In our study, FXR activation decreased TNFα levels and the degree of fibrosis in I/R injured kidneys. Thus, it is possible that the 6-ECDCA treated group had fewer damaged nephrons, and that this was reflected in better renal function and preservation of glomerular structure, as compared with the group receiving only I/R.

**Figure 9:**
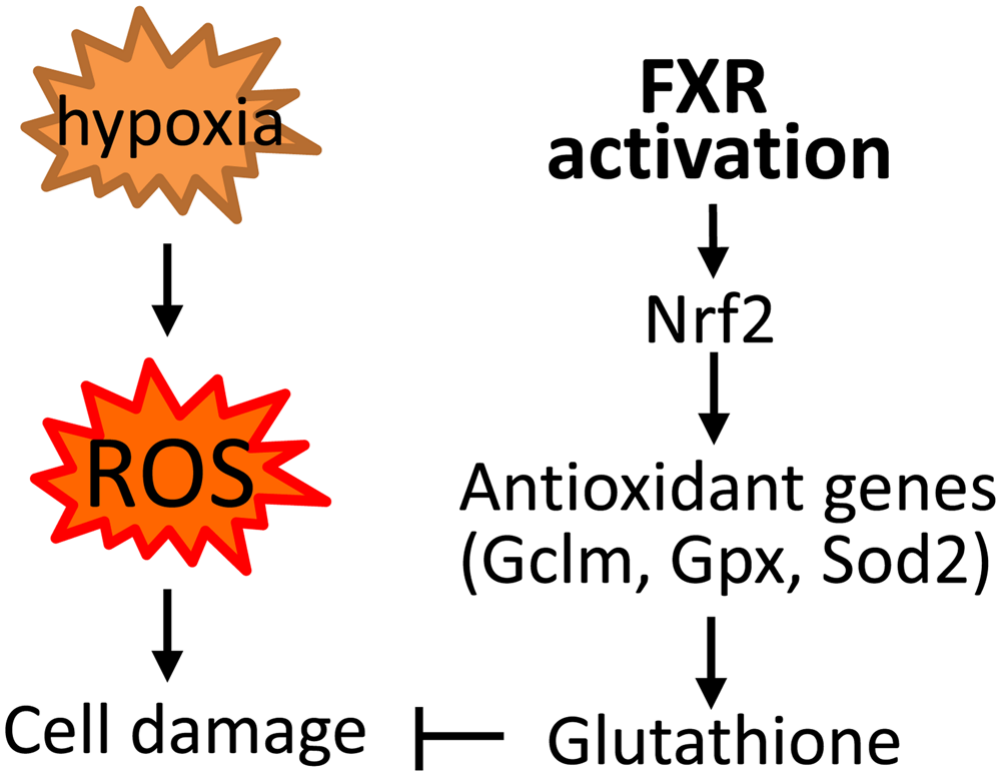
Scheme showing that hypoxia promotes ROS through inducing inflammation and epithelial cell damage. FXR activation inhibits I/R-induced ROS by reducing inflammatory cytokines and inducing antioxidant molecules in the proximal tubule cells.

Previous studies proved a substantial protective role for FXR activation in mitochondrial membrane peroxidation systems, through its antioxidant and free radical-scavenging activities^28, 49–51^. FXR may also protect the mitochondria by interacting with FoxO3a^52^, Nrf2^53^ and AMPK^54^ pathways, all of which are important for mitochondrial function. Our data showed that FXR activation restored the oxidative balance, improved mitochondrial function and reduced ER stress in kidneys subjected to I/R. Moreover, we demonstrated that Nrf2 mediated the protective effects of FXR against oxidative stress. Silencing of Nrf2 blocked the protective effect of 6-ECDCA in proximal tubule cells exposed to hypoxia (Fig. 9). Based on these findings, FXR is a potential target for future therapeutic approaches in patients suffering from kidney ischemia secondary to cardiovascular surgery and kidney transplantation as well as in other kidney impairments.

Sustained leukocyte accumulation and activation inside the kidney could extend periods of ischemia and may induce direct tubular and endothelial cell damage by the release of ROS. Although the impact of FXR activation on leukocyte infiltration in the I/R kidney was not explored in this study, several lines of evidence indicate that FXR activation could also reduce ROS through regulating leukocyte activation. Several studies showed that FXR has anti-inflammatory properties and that these properties are potentially beneficial for treating various inflammatory diseases. The first such evidence was derived from the observation that FXR inhibited NFκB, the downstream pathway regulated by TNFα^55^. FXR activation reduced hepatic inflammation and fibrosis in a mouse model of non-alcoholic steatohepatitis^56^, whereas FXR deficiency caused increased hepatic inflammation and fibrosis^57^. FXR also inhibited expression of profibrotic growth factors and proinflammatory cytokines in both *in vivo* and *in vitro* studies^40^, consistent with our findings. Moreover, a recent study demonstrates that dual FXR and TGF5 activation induces the transition of macrophages from Ly6Chigh into Ly6Clow phenotype and increases IL-10 production^58^, indicating potential regulatory effects of FXR on macrophage activation and release of ROS (Fig. 8K).

In conclusion, this study illustrates that the damage incurred by the kidney after an I/R episode leads to increased ROS levels and associated mitochondrial and ER stress. Activation of the bile acid receptor FXR reduces renal inflammation and apoptosis, lowers ROS levels, and protects kidney mitochondria in mice subjected to I/R. Moreover, FXR activation prevents oxidative stress-induced mitochondrial damage by upregulating antioxidant pathways and controlling glutathione metabolism, providing several likely explanations for its beneficial effect.

## Methods

### Animals

Female C57/BJ mice, aged 6 wk, were randomly assigned to I/R or sham procedures. They were divided into 3 groups of 6 animals each: sham, I/R and I/R + ECDCA. AKI was induced using unilateral with contralateral nephrectomy, as previously described^59^. For 6-ECDCA pretreatment, mice were injected intraperitoneally (i.p.) with 6-ECDCA (5 mg/kg, ab144246, Abcam, Cambridge, UK) 24 h before ischemia. In another set of experiments, mice received 6-ECDCA (5 mg/kg, i.p.) at 8 or 2 h before ischemia or 30 min after I/R. To model I/R induced CKD, mice were maintained in their housing cages and fed a normal diet for 16 wk. All mice were killed under anesthesia at different times after surgery. Kidneys were harvested for further analysis.

### Measurements in plasma and urine samples

Urinary albumin and creatinine concentrations were measured in the resulting urine samples with the albumin mouse ELISA kit (ab108792, Abcam, Cambridge, UK) and creatinine assay kit (ab65340, Abcam), respectively. Urinary H_2_O_2_ levels were measured using the Amplex Red H_2_O_2_ assay kit (A12214, Invitrogen, CA, USA). Plasma creatinine and blood urea nitrogen (BUN) levels were measured with a creatinine assay kit (ab65340, Abcam) and the QuantiChrom™ Urea Assay Kit (DIUR-100, BioAssay System, Hayward, CA, USA), respectively.

### Renal pathological assessments and immunostaining

Tissue sections were stained with hematoxylin and eosin (HE), Periodic acid-Schiff’s (PAS) and Masson’s trichrome stains using standard protocols. The mesangial area was determined from the PAS stained sections and the fibrotic area from the Masson’s trichrome stained sections as previously described^28^. The antibodies used in this study were those against FXR (sc-13063, Santa Cruz), Grp78 (ab21685, Abcam), Chop (ab59396, Abcam), 4-hydroxynonenal (4-HNE, ab46545, Abcam), Kim-1 (ab78494, Abcam), 8-oxo-2′-deoxyguanosine (8-oxo-dG, ab64548, Abcam), SDHA (#11998, CellSignaling, Danvers, MA, USA), tumor necrosis factor (TNF, ab6671, Abcam), CD4 (sc-7219, Santa Cruz, Dallas, TX, USA), aSMA (NBP1-30894, Novus Biologicals, Littleton, CO, USA) and Collagen I (ColI, NB600-408, Novus Biologicals). TUNEL staining on kidney paraffin sections was performed with an ApopTag kit (Millipore, Billerica, MA, USA), according to the manufacturer’s instructions.

### ROS determination in kidneys

Fresh renal cortex tissue slices, approximately 200 μm thick, were cut with a blade and placed into Hanks solution containing 10 mM Hepes-NaOH, pH 7.4 and the fluorescent ROS probe. Kidney slices were loaded with 10 μM 2′,7′-dichlorodihydrofluorescein diacetate (2′,7′-DCFDA, Molecular Probes, Inc., Eugene, Oregon, USA) for 10 min at room temperature followed by a 5 min wash in Hanks solution. A threshold series of images was processed with ImageJ software (National Institutes of Health, Bethesda, MD, USA) to determine relative intensities of 2′,7′-dichlorohydrofluorescein (2′,7′-DCF).

### Isolation of primary proximal tubular cells and cell staining

Primary proximal tubular cells were isolated and stained as described previously^28^. After treatments, cells were fixed and incubated with primary antibodies, as indicated, at 4 °C overnight. The cells were then washed and incubated with the appropriate secondary antibody for 1 h in the dark. Antibodies against Grp78 were purchased from Abcam. For intracellular ROS detection (CellRox, Life Technologies), mitochondrial membrane potential (∆Ψm) detection (TMRM, Molecular Probes), glutathione detection (ThioTracker Violet, Life Technologies), and TUNEL staining (ApopTag, Millipore), cells were processed according to the manufacturer’s protocols. Cells were analyzed for malonyldialdehyde (MDA) (ab118970; Abcam), ATP (CellTiter-Glo, Promega) and GSH (ApoGSH, BioVision) using specific assay kits, according to the manufacturers’ instructions.

### Hypoxia/Re-normoxia model in proximal tubular cells

Prior to the procedure, cells were cultured to 70%–80% confluence, then serum deprived overnight. Next the effects of hypoxia on oxidative and ER stress in primary cultured cells were investigated under low oxygen (1% O_2_) for 12 h. Where indicated, cells were pretreated with 1 μM GW4064 or 1 μM 6-ECDCA for 4 h prior to hypoxia treatment. For the RNA interference experiment, cells were pretreated with Nrf2 or FXR siRNA (100 nM) for 24 hours before exposure to hypoxia.

### Isolation of RNA from kidney tissue and cells and quantification of transcript levels

Total RNA was prepared using Trizol (Invitrogen, CA, USA). The mRNA was quantified based on absorbance at 260 nm. After DNAse (Promega) treatment, 2 μg total RNA was reverse transcribed using oligo-dT priming and Superscript II (Invitrogen). First-strand complementary DNA was used as the template for real-time polymerase chain reaction analysis with TaqMan master mix and primers (Applied Biosystems, CA, USA). Transcript levels, determined in two independent complementary DNA preparations, were calculated and expressed relative to levels of RNA for the housekeeping gene villin.

### Western blotting

Protein lysates (20 μg protein) of kidney tissue or cells were separated by SDS-PAGE and blotted on polyvinylidene difluoride membranes (Millipore). The membranes were incubated overnight at 4 °C with the respective primary antibodies and secondary antibodies accordingly. Staining was then developed using the ECL Plus detection system (Amersham Biosciences, Little Chalfont, UK). The antibody against villin was from Chemicom (MAB1639a, Toronto, Canada), anti-tubulin (T6074) was from Sigma-Aldrich (Buchs, Switzerland), anti-Grp78 (ab21685) was from Abcam, anti-ICAM (sc-1511), anti-FXR (sc-13063) and anti-Nrf2 (sc-722) were from Santa Cruz and anti-SDHA (#11998) and anti- cleaved caspase 3 (#9661) were from CellSignaling (Danvers, MA, USA).

### Study approval

All animal experiments conformed to both Swiss and Chinese animal protection laws and were approved by the Scientific Animal Study Committee of Shandong University, Jinan, China (study number 2015064). The human study was conducted according to the Declaration of Helsinki guidelines regarding ethical principles for medical research involving human subjects. All patients provided written informed consent and the study protocol was approved by the Scientific Ethical Committee of Shandong University, Jinan, China, where the patients were based (license number SDU2007011 and SDU2015033).

### Statistics

Data are expressed as means ± SEM. For data relating to baseline characteristics and histological analysis, groups were compared by one-way ANOVA followed by Bonferroni’s test. For data relating to demographics and clinical characteristics, groups were compared by one-way ANOVA followed by Bonferroni’s test or by Mann–Whitney U test. Statistical analyses were performed using GraphPad software (CA, USA).

## Electronic supplementary material

Supplementary Methods, Table and Figures
