# Supplementary material for: Farnesoid X receptor activation protects the kidney from ischemia-reperfusion damage
Source: Sci Rep. 2017 Aug 29;7:9815. doi: 10.1038/s41598-017-10168-6 (PMC5575310; doi:10.1038/s41598-017-10168-6)
Supplement: Supplementary file 1 — Supplementary Methods, Table and Figures [file 41598_2017_10168_MOESM1_ESM.pdf]

Farnesoid X receptor activation protects the kidney from ischemia-reperfusion damage

Zhibo Gai[1§], Lei Chu [2§], Zhenqiang Xu [3], Xiaoming Song[4], Dongfeng Sun[4*] and Gerd A. Kullak-Ublick[1*]

[1]Department of Clinical Pharmacology and Toxicology, University Hospital Zurich, University of Zurich, Switzerland
[2]Department of Urology, Tengzhou Central People's Hospital, Zaozhuang, People's Republic of China
[3]Department of Cardiovascular Surgery, Shandong Provincial Hospital affiliated to Shandong University, Jinan, People's Republic of China
[4] Department of Thoracic Surgery, Shandong Provincial Qianfoshan Hospital, Shandong University, Jinan, People's Republic of China

To whom correspondence should be addressed: Gerd A. Kullak-Ublick, M.D. Department of Clinical Pharmacology and Toxicology, University Hospital Zurich, Rämistrasse 100, CH-8091 Zurich, Switzerland, Telephone: + 41 44 255 2068; FAX: + 41 44 255 9676; E-mail: gerd.kullak@usz.ch; or Dongfeng Sun, M.D. Department of Thoracic Surgery, Shandong Provincial Qianfoshan Hospital, Shandong University, Jinan, People's Republic of China, 250014. E-mail: sundongfeng81@gmail.com

Methods

Renal histological analysis

Histopathologic damage was defined as tubular epithelial swelling, loss of brush border, vacuolar degeneration, necrotic tubules, cast formation and desquamation. The degree of tubular injury was estimated at a 200× magnification analyzing 5 randomly selected fields for each kidney and using the following scoring system: 0, normal; 1, damage involving <25% of tubules; 2, damage involving 25%–50% of tubules; 3, damage involving 50%–75% of tubules; and 4, damage involving 75%–100% of tubules.

Patients

Kidney preimplantation biopsies, 17 in total, were obtained from deceased kidney donors in the Provincial Hospital Affiliated to Shandong University from 1 Jan 2014 to 30 Dec 2015. Data were obtained retrospectively through systematic review of medical charts and the electronic database. Patients with a history of hypertension, diabetes, proteinuria, use of nephrotoxic drugs or with serum creatinine levels of >106 μmol/l before surgery were excluded.

For the longer I/R study, clinical information was collected from patients undergoing cardiac surgery from 1 Jan 2007 to 30 Dec 2011 in the Provincial Hospital Affiliated with Shandong University. A total of 3279 cases of cardiopulmonary bypass were reviewed. Among these cases, 256 patients had AKI after cardiopulmonary bypass. AKI was defined as a urine volume of <0.5ml/kg/h for more than 6 h. Of these 256 patients, 25 had developed CKD by 30 Dec 2015. Patients with hypertension, endocarditis, diabetes, proteinuria or serum creatinine levels of >106 μmol/l before surgery were excluded. During this period, there was no history of nephrotoxic drug use by any patient, except for anticoagulatory agents and diuretics as required. CKD after cardiac surgery was diagnosed according to the KDIGO Clinical Practice Guideline for the Evaluation of Chronic Kidney Disease. Kidney biopsies from patients with CKD were collected by ultrasonography-guided percutaneous biopsy.

The control biopsies were renal biopsies that appeared to be normal by histological, immunofluorescence and electron microscopic examinations. These controls were obtained either from uninvolved portions of a kidney at the time of tumor nephrectomy or from candidate kidney donors. Demographic data on control and CKD groups, such as gender, age, urinary protein and eGFR are shown in Supplementary Table S1.

Table S1: Demographic and clinical characteristics of AKI and CKD patients

Variable	Normal (n= 20)	AKI (n= 17)	CKD (n= 25)
Gender (male/female)	12/8	10/7	13/12
Age (years)	54.0±8.3	49.1±6.4	56.1±6.2
Serum creatinine before surgery (μmol/L)	76.0±12.8	83.1±16.4	78.1±13.5
Serum creatinine follow-up (μmol/L)	79.6±16.5	N.A.	165.8±35.0*
eGFR follow-up (ml/min/1.73m^2)	113.1±14.7	N.A.	55.7±13.7*
Histology (at the time of biopsy)			
Glomeruli (number)	24.4±8.9	25.7±6.5	26.1±9.0
Glomerulosclerosis (%)	1.5±1.3	2.3±1.0	29.5±9.8*
Mesangial matrix	0.4±0.3	1.1±0.1	2.1±0.4*
Interstitial fibrosis (%)	3.6±0.4	2.2±0.1	11.3±0.8*

* p<0.05 compared with Normal group. Data are means ± SEM.

The degree of mesangial matrix was evaluated based on an arbitrary scale: 0=normal; 1=focal; 2=increased, mild; 3=increased, nodular. Fibrosis was evaluated based on Masson's trichrome staining and glomerulosclerosis was evaluated based on PAS staining. All analyses were taken from routine pathological evaluations.

Abbreviations: AKI, acute kidney injury; CKD, chronic kidney disease; eGFR, estimated glomerular filtration rate

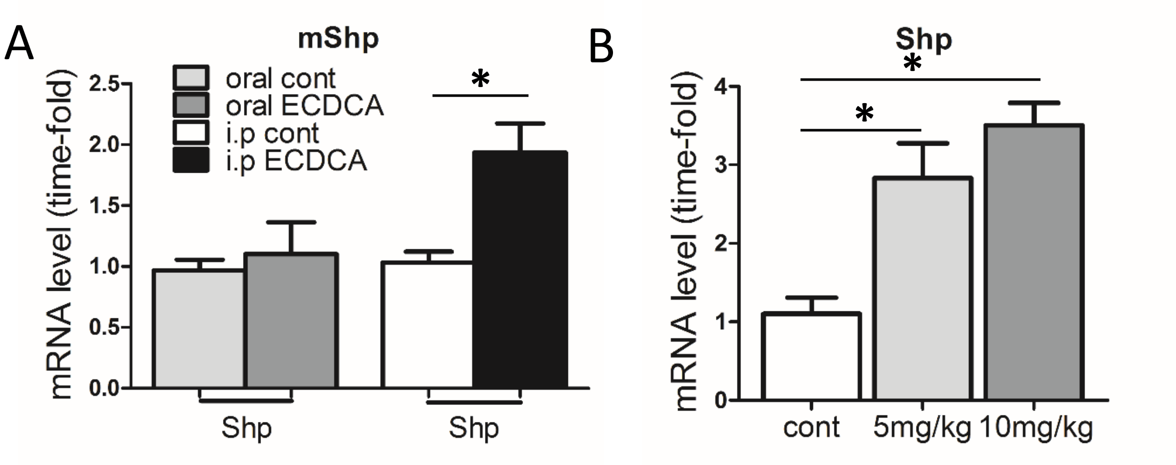

Supplementary Figure S1 Kidney FXR activation by different 6-ECDCA treatments in mice. (A) Relative induction of FXR target gene Shp in kidney after a single i.p. injection or a single oral gavage at the same dosage (5mg/kg) of 6-ECDCA, compared with vehicle. (B) Relative induction of the FXR target gene Shp in kidney after a single i.p. injection of 6-ECDCA at different dosages, compared with vehicle. Data are mean ± SEM. Statistical analysis was done using Student's t-test. *$p<0.05$. n=3-5 per group.

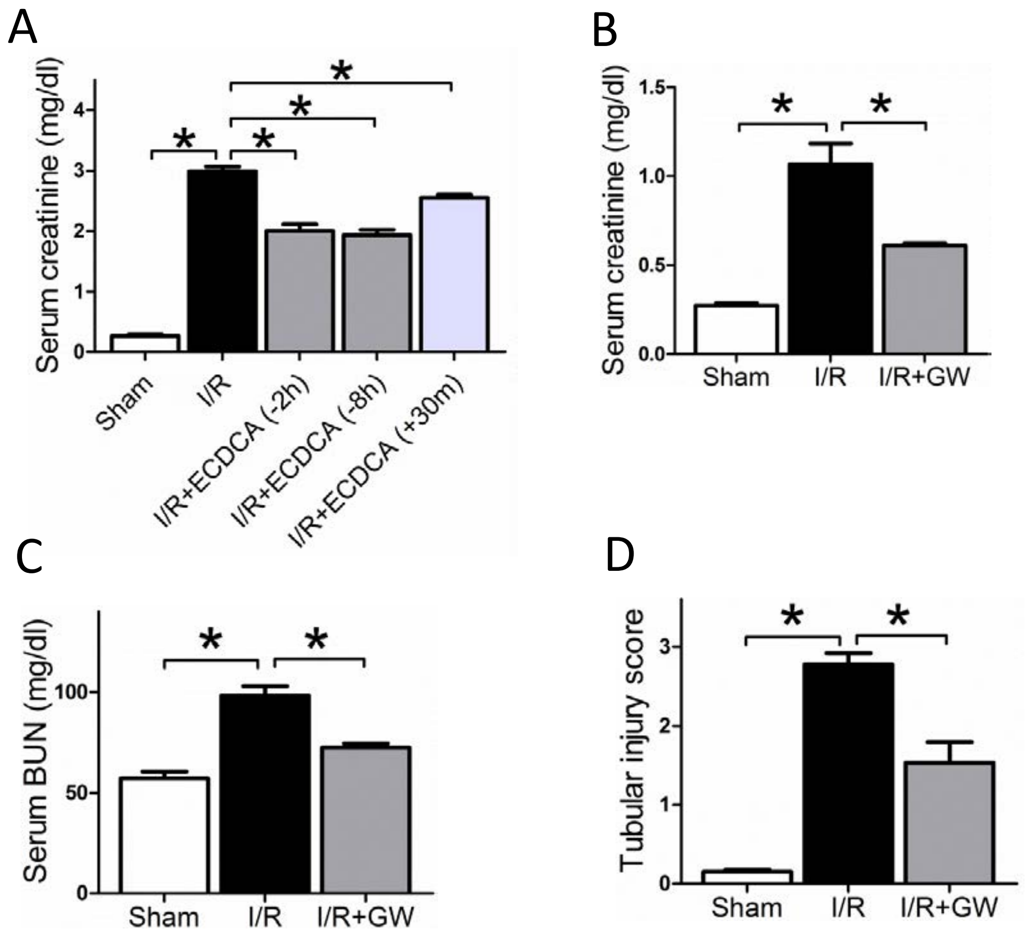

Supplementary Figure S2 FXR activation ameliorates kidney function after I/R. (A) Mice were injected i.p. with 6-ECDCA at different timepoints in relation to I/R, as indicated. At 24 h after I/R, serum samples were collected for measurement of creatinine. *n*=6 mice/group. (B to D) Mice were injected i.p. with GW4064 before I/R injury. At 24 h after I/R, serum samples were collected for measurement of (B) BUN and (C) creatinine. (D) Quantitative analysis of tubular injury scores. *n*=6 mice/group.

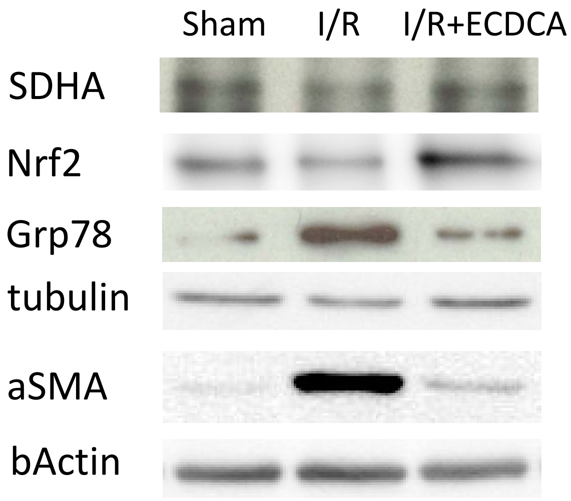

Supplementary Figure S3 Representative western blot images of SDHA, nuclear Nrf2, Grp78 and aSMA in kidney samples from sham, I/R and I/R+ECDCA groups.

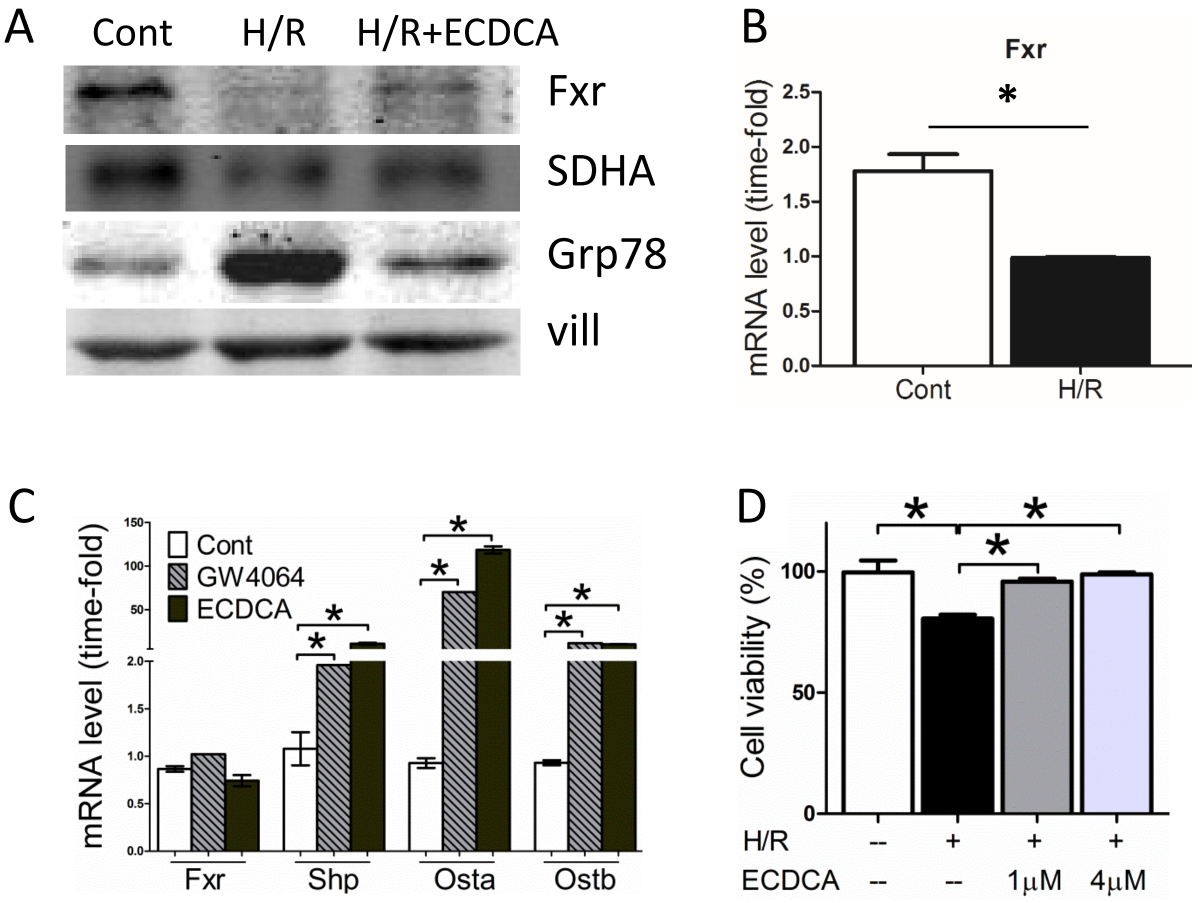

Supplementary Figure S4 6-ECDCA activates FXR in PTCs and protects cells from hypoxia-induced damage (A) Representative western blots of Fxr, SDHA and Grp78 in cell lysates of PTCs. (B) Quantification of FXR mRNA level in PTCs (C) mRNA analysis of genes downstream to FXR activation in PTCs after 6-ECDCA treatment, demonstrating that 6-ECDCA successfully activated FXR in PTCs. (D) Cell viability analysis of PTCs. n=4/group. Data are means ± SEM, one-way ANOVA with Bonferroni's test. *, $p<0.05$.

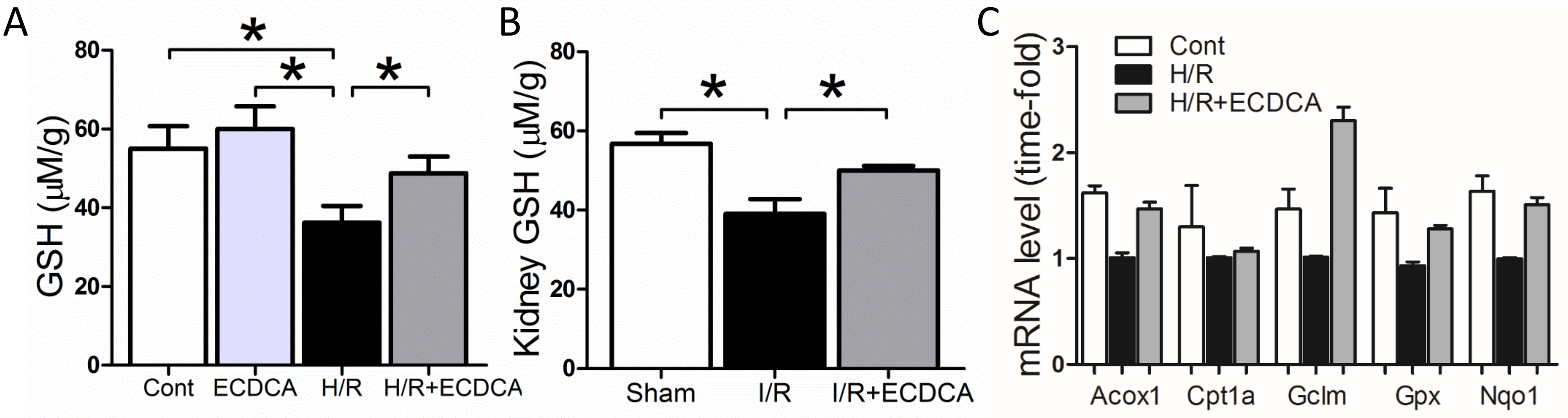

Supplementary Figure S5 6-ECDCA induces GSH in PTCs and kidney (A and B) Quantification of GSH levels from (A) PTCs or (B) kidneys. (C) Quantification of mRNA levels of genes associated with mitochondrial function (Acox1, Cpt1), glutathione metabolism (Gclm, Gpx) and anti-oxidation (Nqo1) from PTCs. *n*=4/group. Data are means ± SEM, one-way ANOVA with Bonferroni's test. *, $p<0.05$.

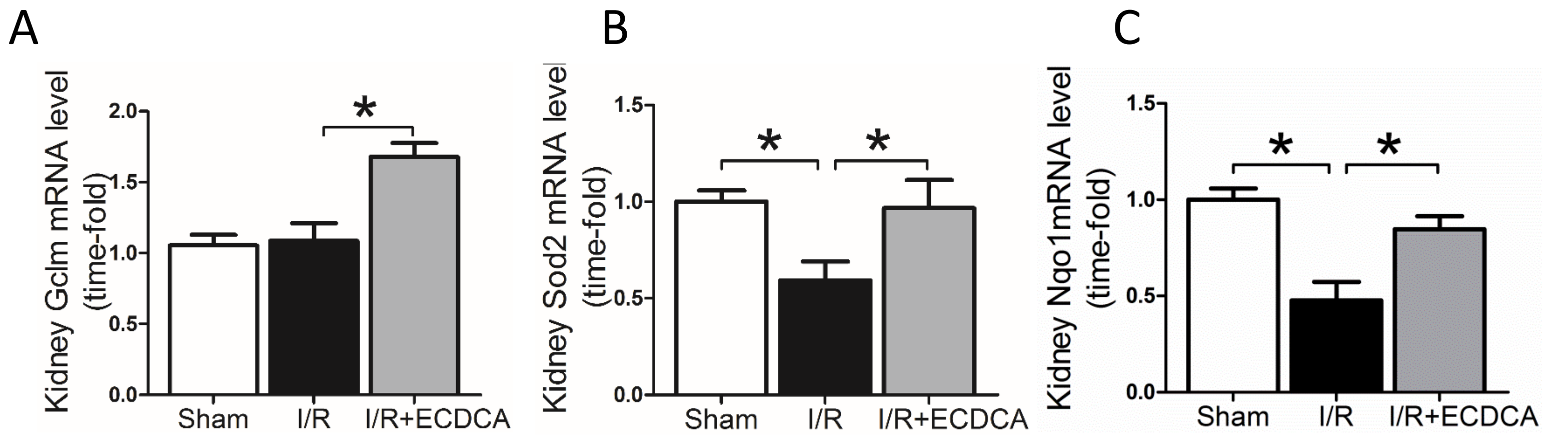

Supplementary Figure S6 FXR activation induces antioxidant genes in kidney (A to C) Quantification of mRNA levels of (A) Gclm, (B) Sod2 and (C) Nqo1 in kidneys from different treatments. n=6/group. Data are means ± SEM, one-way ANOVA with Boferroni's test. *, p<0.05.

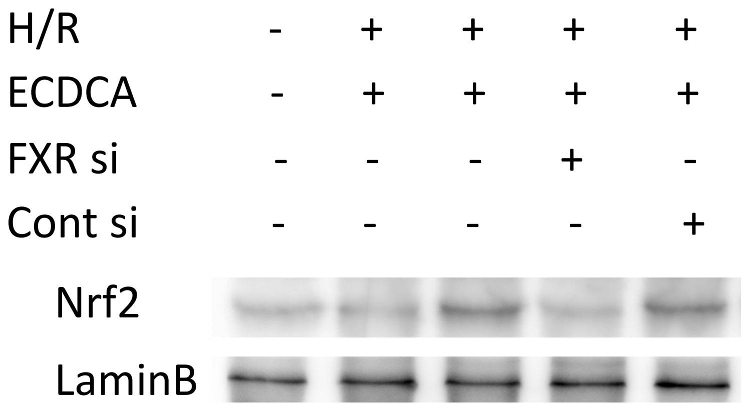

Supplementary Figure S7. Silencing of FXR by siRNA abolishes the inductive effect of ECDCA on nuclei Nrf2 protein levels. Representative western blot images of Nrf2 and Lamin B1 in cell nuclear extracts from PTCs treated as indicated.

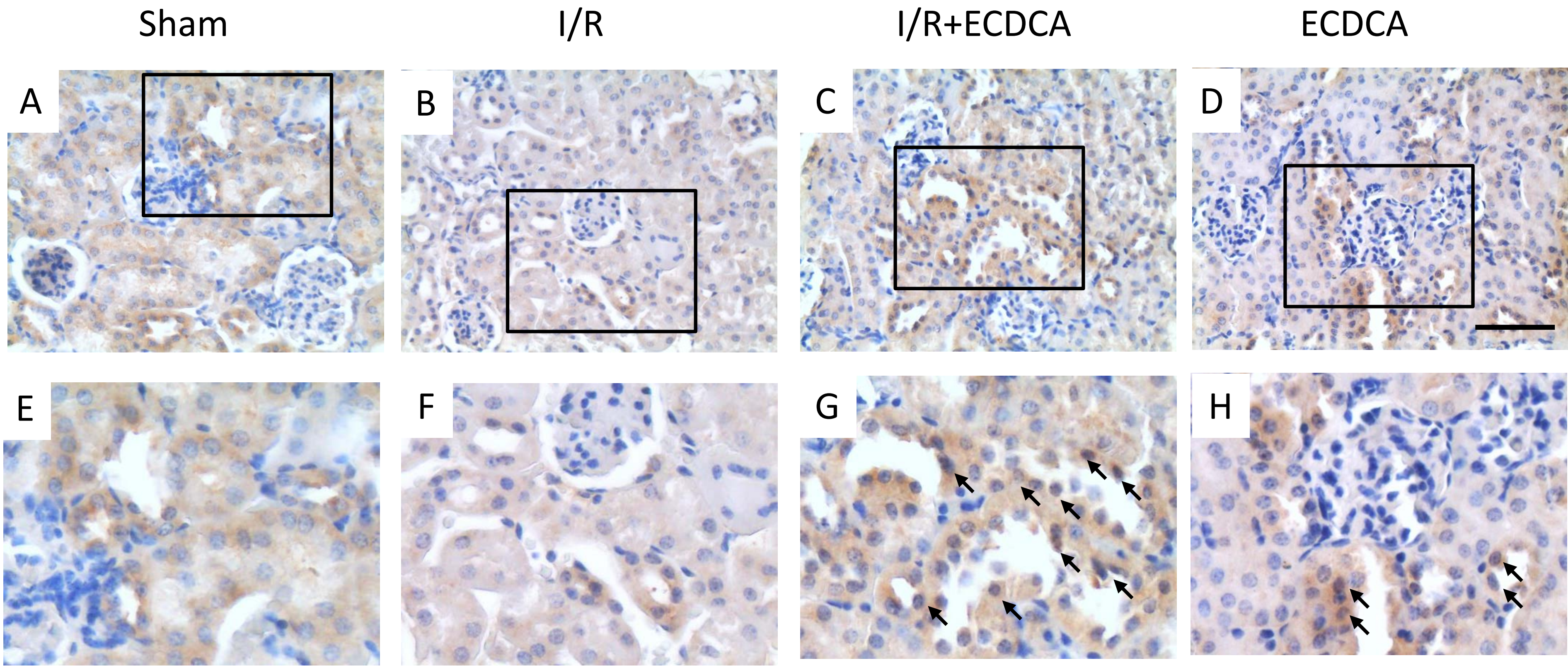

Supplementary Figure S8. Representative images showing HE staining on renal sections from (A) sham, (B) I/R, (C) I/R+ECDCA and (D) ECDCA treatment alone groups (scale bar 50μm). (E to H) The areas delineated by rectangles in the top panels are shown at higher magnification in the bottom panels. Arrows indicate positive nuclei staining of Nrf2.

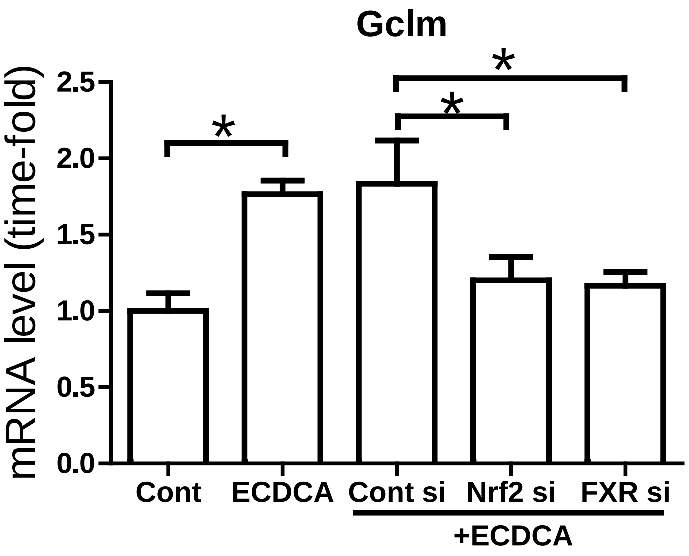

Supplementary Figure S9. Quantification of mRNA levels of the Nrf2 target gene, Gclm, in primary cultured renal proximal tubular cells. Silencing of Nrf2 or FXR by siRNA abolishes the inductive effect of ECDCA on Gclm mRNA levels. *n*=4/group. Data are means ± SEM, one-way ANOVA with Bonferroni's test. *, *p*<0.05.
